# Mortality among amphetamine users with hepatitis C virus infection: A nationwide study

**DOI:** 10.1371/journal.pone.0253710

**Published:** 2021-06-24

**Authors:** Caroline Gahrton, Anders Håkansson, Martin Kåberg, Anna Jerkeman, Henrike Häbel, Olav Dalgard, Ann-Sofi Duberg, Soo Aleman

**Affiliations:** 1 Division of Infection and Dermatology, Department of Medicine Huddinge, Karolinska Institute, Stockholm, Sweden; 2 Department of Infectious Diseases, Karolinska University Hospital, Stockholm, Sweden; 3 Faculty of Medicine, Department of Clinical Sciences Lund, Lund University, Psychiatry, Lund, Sweden; 4 Region Skåne, Malmö Addiction Center, Clinical Research Unit, Malmö, Sweden; 5 Faculty of Medicine, Department of Translational Medicine, Clinical Infection Medicine, Lund University, Malmö, Sweden; 6 Division of Biostatistics, Institute for Environmental Medicine, Karolinska Institutet, Stockholm, Sweden; 7 Department of Infectious Diseases, Akershus University Hospital, Lørenskog, Norway; 8 Institute of Clinical Medicine, University of Oslo, Oslo, Norway; 9 Faculty of Medicine and Health, Department of Infectious Diseases, Örebro University, Örebro, Sweden; Kaohsiung Medical University Chung Ho Memorial Hospital, TAIWAN

## Abstract

**Aims:**

To investigate liver-related and all-cause mortality among amphetamine users with hepatitis C virus (HCV) infection and compare this with opioid users with HCV infection and the uninfected general population.

**Methods:**

In this national register study of mortality in persons notified with HCV infection 1990–2015 and a substance-related diagnosis in Sweden, amphetamine users (n = 6,509) were compared with opioid users (n = 5,739) and a matched comparison group without HCV infection/substance use (n = 152,086).

**Results:**

Amphetamine users were observed for 91,000 years and 30.1% deceased. Crude liver-related mortality was 1.8 times higher in amphetamine users than opioid users (crude mortality rate ratio 1.78, 95% CI 1.45–2.19), but there was no significant difference when adjusting for age and other defined risk factors. An alcohol-related diagnosis was associated with liver-related death and was more common among amphetamine users. Crude and adjusted liver-related mortality was 39.4 and 5.8 times higher, respectively, compared with the uninfected group. All-cause mortality was lower than in opioid users (adjusted mortality rate ratio 0.78, 95% CI 0.73–0.84), but high compared with the uninfected group. External causes of death dominated in younger ages whereas liver-related death was more common among older individuals.

**Conclusions:**

This national register study presents a higher crude risk of liver-related death among HCV-infected amphetamine users compared with opioid users or the uninfected general population. The higher risk of liver-related death compared with opioid users may be explained by lower competing death risk and higher alcohol consumption. Treatment of HCV infection and alcohol use disorders are needed to reduce the high liver-related mortality.

## Introduction

Chronic hepatitis C virus (HCV) infection cause significant morbidity and mortality globally [1]. Within 20 years of infection, approximately 20% develop liver cirrhosis with an increased risk of hepatocellular carcinoma (HCC) and decompensated liver disease [2–4]. The World Health Organization (WHO) has therefore set a goal to eliminate HCV infection as a public health threat, through the reduction of mortality by 65% and new infections by 80% by the year 2030, compared to 2015 levels [5].

An important route of HCV infection is injection drug use. Consequently, there is a high prevalence of HCV among people who inject drugs (PWID) with 48.5% estimated prevalence worldwide, or 5.5 million people [6]. Stimulants are commonly used among PWID and constitute the primary drug used in 33% globally [7]. In Sweden, the HCV prevalence among PWID is as high as 62.1%, and amphetamine is the most commonly injected drug [8].

Globally it is estimated that 585,000 persons died in 2017 as a result of drug use [6]. People with amphetamine use have increased mortality compared with the general population, mainly from directly drug-related death, but also due to suicide, homicide, cardiovascular disease, and injuries [9]. Thus, HCV-infected PWID have an increased risk of both drug-related and liver-related morbidity and death. Previous studies of HCV-infected PWID reported that substance use disorders dominate as cause of death among younger persons, but the risk of liver-related death increases with age and is one of the main causes of death among older PWID [10,11].

There are studies reporting overall mortality of people who use drugs [9,12], but data on HCV-infected amphetamine users are scarce and large cohort studies are missing. Compared with opioid users, life expectancy is higher in amphetamine users, probably due to the higher lethal risk of opioid intoxication compared with what is seen from stimulants like amphetamine [9,12–14], which might affect the risk of liver-related death. In addition, amphetamine users may have less contact with health care providers than opioid users due to the lack of substitution treatment, which might result in a lower uptake to HCV treatment and further increase the burden of HCV disease. The prevalence of harmful alcohol consumption may also differ between different groups of PWID [15]. Therefore, we aimed to investigate liver-related and all-cause mortality among HCV-infected people who use amphetamine compared with HCV-infected people who use opioids and with the uninfected general population.

## Methods

### Study population and registers

In this cohort study, with an external comparison cohort, we used prospectively collected data from national registers. The study population consisted of all persons in Sweden notified with an HCV infection to the Public Health Agency year 1990–2015, and with an amphetamine or opioid diagnosis in the National Patient Register (NPR) at any time point from Jan 1^st^ 1987 -Dec 31^st^ 2017 [16]. The Public Health Agency administers the national registers of HCV and HBV infections, to which it is mandatory to report all new infections by both laboratories and clinicians. The HCV notification is based on anti-HCV or HCV RNA, constituting either an acute or chronic infection.

All residents in Sweden have a unique personal identification number (PIN) that is used when in contact with the health care system and for national registers. These PINs were used to link individual information from the national registers.

The HCV notification data were transferred to Statistics Sweden where PINs were checked, residence in Sweden was confirmed for the study, and information on emigration and date of death was obtained. A comparison cohort without HCV infection, referred to as controls in this manuscript, was produced by Statistics Sweden, where each HCV-infected person was matched with 10 randomly selected persons without an HCV diagnosis from the general population. The matching criteria were birth year, sex and county of residence, and the matching date was the date of HCV notification.

Thereafter, the data were transferred to the National Board of Health and Welfare, where information on substance use and risk factors was retrieved from the NPR. The NPR contains the dates and diagnoses relating to all inpatient hospital admissions since 1987 and all outpatient specialist care since 2001. The diagnoses are classified according to the WHO International Statistical Classification of Diseases and Related Health Problems (ICD)-9, year 1987–1996, and ICD-10, year 1997-. All ICD codes used for this study are provided in the supplementary material (S1 Table).

By using the NPR, the HCV-infected study population with substance use-related diagnoses was identified into the following groups: amphetamine diagnosis only, opioid diagnosis only, and both amphetamine and opioid diagnosis (named as amphetamine-opioid users in this study). A person with an amphetamine and/or opioid use diagnosis was considered a PWID from the HCV notification date even when the person received a substance use-related diagnosis after notification, since HCV usually is contracted through injection drug use. Persons with a liver transplantation diagnosis registered in the NPR (ICD-9 or ICD-10) before the start of observation and persons with an HBV infection notified to the Public Health Agency of Sweden 1990–2015 were excluded. Persons with opioid or amphetamine diagnosis in the NPR were excluded from the controls.

**Fig 1:**
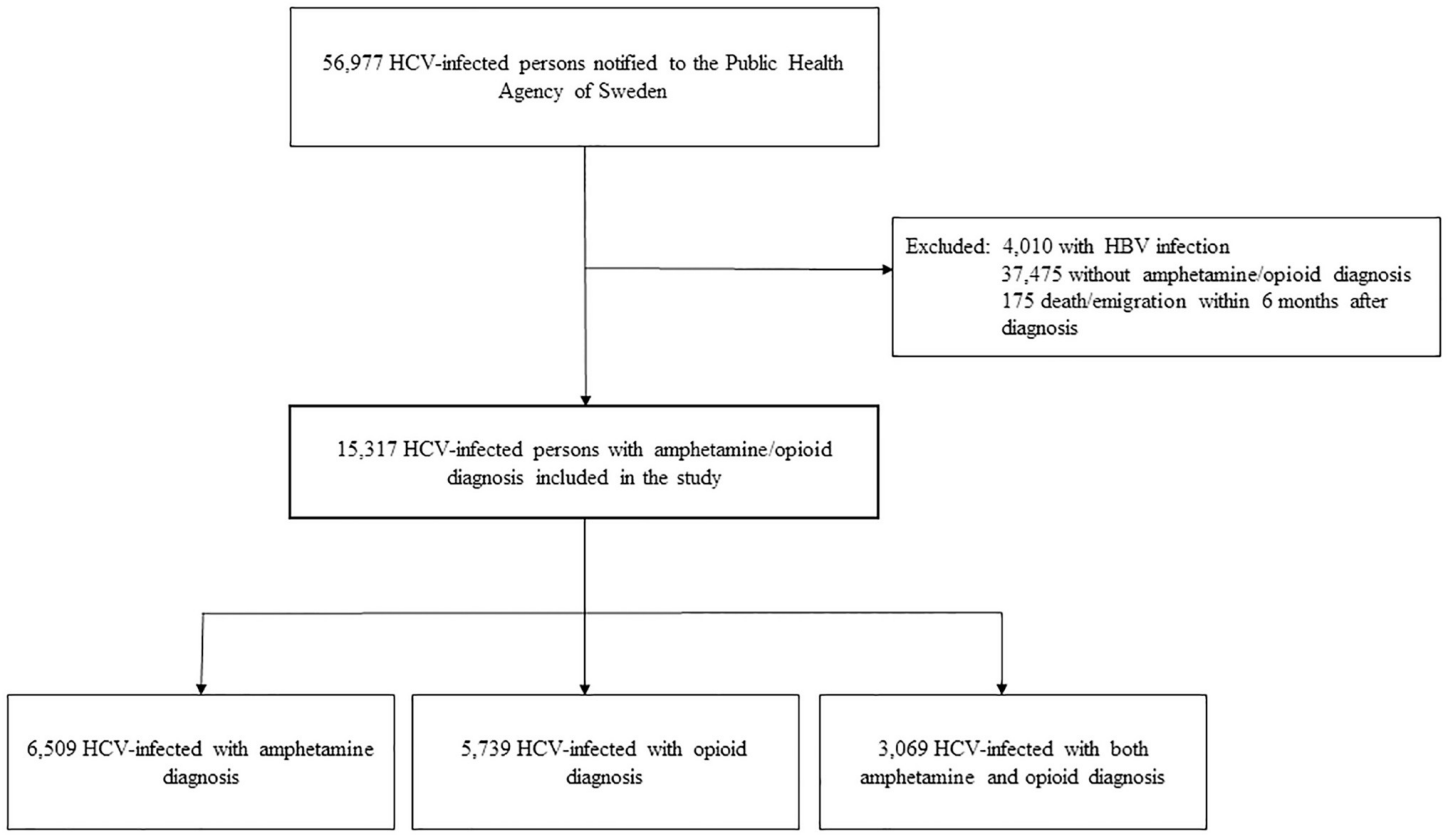
Study population.

Using the above-mentioned procedures, 56,977 persons with HCV infection 1990–2015 were identified from the Public Health Agency (Fig 1). After the exclusion of 4,010 persons co-infected with HBV, 37,475 persons without an amphetamine/opioid diagnosis, and 175 persons who died or emigrated within 6 months after their HCV notification, there were 15,317 HCV-infected persons with an amphetamine/opioid diagnosis included in the study. The controls from the general population consisted of 152,086 individuals without HCV infection/substance use.

### Outcomes

The primary outcomes were liver-related and all-cause mortality. In addition, death from mental and behavioural disorders, external causes (including intoxications, suicide, accidents, and homicide), drug- and alcohol-related death, and death from infections (including hepatitis and HIV), neoplasms, diabetes mellitus, the circulatory system, the respiratory system, and the digestive tract were assessed. The National Board of Health and Welfare provided information from the Cause of Death Register (DR), which includes causes of death since the year 1961 [17], classified according to ICD-9, years 1987–1996, and ICD-10, years 1997-. Outcomes were analyzed as underlying cause of death except for the variables of alcohol- and drug-related death. These were predefined by the National Board of Health and Welfare and based on multiple causes of death, including all causes of death mentioned on the death certificate, with underlying and contributing causes of death. Drug-related death includes death from amphetamine disorders, opioid disorders, and other substance-related disorders as specified in the S1 Table.

### HCV treatment uptake and risk factors

The National Board of Health and Welfare also provided information from the Swedish Prescribed Drug Register (SPDR). The SPDR started July 1^st^ 2005 and includes all prescribed drugs dispensed at pharmacies in Sweden, classified according to the Anatomical Therapeutic Chemical (ATC) classification system. Hepatitis C virus treatment uptake was defined as the proportion of persons with dispensed HCV treatment (interferon or direct-acting antiviral (DAA) therapy) from July 1^st^ 2005, when the SPDR started, until end of follow-up, among all persons who were still alive and were living in Sweden on July 1^st^ 2005. Treatment uptake of DAA was defined as the proportion of persons with dispensed DAA therapy Jan 1^st^ 2014-Dec 31^st^ 2017.

Alcohol-related diagnosis, diabetes mellitus, and HIV were assessed as risk factors of all outcomes. Alcohol-related diagnosis was defined as an alcohol-related diagnosis in the NPR or the DR Jan 1^st^ 1987- Dec 31^st^ 2017. Diabetes mellitus was defined as one ATC code of diabetes mellitus medication (A10.x) in the SPDR, at any point of time from July 1^st^ 2005- Dec 31^st^ 2017, or a diagnosis in the NPR during follow-up. Diabetes mellitus and HIV were analyzed as time-dependent variables, whereas alcohol use was not, as it was considered to be an indicator of a long-standing condition. HIV was defined as at least two diagnoses in the NPR and/or DR.

### Ethics

The Public Health Agency of Sweden, Statistics Sweden, and the National Board of Health and Welfare handled all linkage of data. Only anonymized data were sent to the researchers. No written informed consent was collected. This study was approved by the Ethical Review Board of Stockholm (Ref: 2015/1282-31 and 2019–01972).

### Statistical analyses

Baseline was set to six months (183 days) after HCV notification to the Public Health Agency to avoid overestimation of the mortality risk due to surveillance bias (for deaths within 6 months after notification, HCV was likely diagnosed as a result of the illness that led to death). Patients were followed until death, or censored at emigration, liver transplantation, or Dec 31^st^ 2017.

Statistical differences between frequencies of risk factors between groups were investigated using the Chi-squared test. A p-value below 0.05 was considered statistically significant. Mortality rates in amphetamine users were compared with the rates in opioid users, amphetamine-opioid users, and controls. All-cause and cause-specific mortality were analyzed using population-averaged Poisson regression models, both unadjusted and adjusted for age, sex, diabetes mellitus, HIV, and alcohol-related diagnosis. Age was included as a time-varying categorical variable with six levels (0–29 years; 30–39 years; 40–49 years; 50–59 years; 60–69 years; ≥70 years). Sex and an alcohol-related diagnosis were included as binary variables. Diabetes mellitus and HIV were also included as binary variables but were allowed to vary across time. The person-time spent in each age group was included as an offset term and the so-called clock-reset approach was applied [18]. Mortality rates (MR) were reported per 100 person-years (PY). The robust sandwich estimator was used to estimate standard errors. Mortality rate ratios (MRR) and 95% confidence intervals (CI) were reported. Crude and adjusted MRRs were estimated for each age group in subgroup analyses using Poisson regression models. In order to test for differences between the MRRs in different age groups population-averaged Poisson regression models including an interaction term between drug user groups and age groups were fitted and Wald tests for the interaction terms were conducted. Analyses were performed using Stata version 15.1 (StataCorp, College Station, TX, USA).

## Results

### Patient characteristics

The study population consisted of 15,317 HCV-infected persons with a substance use-related diagnosis, of which 6,509 (42.5%) had an amphetamine diagnosis, 5,739 (37.4%) an opioid diagnosis, and 3,069 (20.0%) an amphetamine and opioid diagnosis (Table 1a). The comparison group consisted of 152,086 people. The percentage of men was 70.5% among amphetamine users, 73.5% among opioid users, 68.8% among amphetamine-opioid users, and 71.2% among controls. The median age at baseline six months after HCV diagnosis was 36, 31, 32, and 33 years for amphetamine users, opioid users, amphetamine-opioid users, and controls, respectively. The total observation time for PWID was 200,796 PY (median 13.3, interquartile range (IQR) 6.8–19.0). The observation times for each group of PWID and controls are presented in Table 1b.

**Table 1:** a. Characteristics of study population by hepatitis C status and substance use (n = 15317). b. Observation time, age at death, and crude all-cause mortality rate by hepatitis C status and substance use.

Characteristics	HCV-infected PWID			Controls without HCV^a^
	Amphetamine users	Opioid users	Amphetamine and opioid users	
Number of persons	6,509	5,739	3,069	152,086
Birth-year, median (IQR)	1963 (1956–1972)	1972 (1961–1981)	1967 (1960–1978)	1966 (1958–1978)
Age at notification/matching date, median (IQR)	36 (29–43)	31 (25–39)	32 (25–38)	33 (26–41)
Male sex, n (%)	4,587 (70.5%)	4,220 (73.5%)	2,110 (68.8%)	108,330 (71.2%)
Education level, n (%)				
≤9 years	3,213 (51.8%)	2,967 (55.3%)	1,703 (58.9%)	25,629 (18.2%)
10–12 years	2,811 (45.3%)	2,174 (40.5%)	1,106 (38.3%)	70,206 (49.7%)
>12 years	182 (2.9%)	225 (4.2%)	82 (2.8%)	45,363 (32.1%)
Country of origin, n (%)				
Nordic	6,294 (96.7%)	4,868 (84.8%)	2,834 (92.3%)	121,605 (80.0%)
Other	215 (3.3%)	871 (15.2%)	235 (7.7%)	30,450 (20.0%)
Alcohol-related diagnosis, n (%)	3,727 (57.3%)	2,117 (36.9%)	1,900 (61.9%)	4,794 (3.2%)
HIV, n (%)	137 (2.1%)	113 (2.0%)	137 (4.5%)	131 (0.1%)
Diabetes mellitus, n (%)	694 (10.7%)	412 (7.2%)	282 (9.2%)	9,022 (5.9%)
HCV treatment, n (%)^b^	1,131 (18.9%)	953 (17.8%)	407 (14.4%)	0 (0.0%)
DAA	509 (8.5%)	476 (8.9%)	204 (7.2%)	
				
PY of follow-up, total	90,996.7	68,536.7	41,262.8	2,307,623.8
PY of follow-up, median (IQR)	14.5 (7.7–20.0)	11.7 (5.9–17.5)	13.9 (7.3–19.2)	16.1 (9.4–20.5)
Deaths n (%)	1,957 (30.1%)	1,567 (27.3%)	983 (32.0%)	6,671 (4.4%)
Age at death, median (IQR)	51.0 (43.0–59.0)	43.0 (33.0–54.0)	46.0 (37.0–53.0)	54.0 (45.0–62.0)
Autopsy performed, n (% of deaths)	1,088 (55.6%)	1,038 (66.2%)	671 (68.3%)	2,688 (40.3%)
All-cause cMR per 100 PY (95% CI)	2.15 (2.06–2.24)	2.29 (2.19–2.39)	2.38 (2.24–2.52)	0.29 (0.28–0.30)

^a^ Without amphetamine/opioid use.

^b^ Proportions out of persons observed from year 2005 when the drug register started: 5983 amphetamine users, 5342 opioid users, and 2836 amphetamine and opioid users.

Abbreviations: HCV, hepatitis C virus; PWID, people who inject drugs; IQR, interquartile range; PY, person-years; cMR, crude mortality rate; CI, confidence interval; DAA, direct-acting antiviral therapy.

An alcohol-related diagnosis was significantly more common among HCV-infected persons with amphetamine use than opioid use (57.3% vs 36.9%) or compared to uninfected controls (57.3% vs 3.2%) (p< 0.001 for both) (Table 1a). Diabetes mellitus was also more common among amphetamine users than opioid users or controls (p<0.001). The presence of HIV infection was not significantly more common in amphetamine users compared with opioid users (p = 0.60) but compared with controls (p<0.001). The frequency of received HCV treatment did not significantly differ between amphetamine and opioid users (p = 0.14).

Overall mortality was high; in total 30.1% of amphetamine users, 27.3% of opioid users, and 32.0% of amphetamine-opioid users were deceased by at the end of the study, to compare with 4.4% of controls. The median age at death was higher among amphetamine users than opioid users (Table 1b). The most frequent underlying causes of death among amphetamine users were external causes (9.0%), circulatory system disorders (4.7%), neoplasms (4.0%) including HCC, digestive tract disorders (2.4%) including liver diseases, and mental and behavioral disorders (2.0%) (Table 2). In total, liver-related deaths constituted 4.5% of all deaths among amphetamine users. External causes were the most common underlying causes of death also among opioid users (12.9%).

**Table 2:** Cause-specific mortality by hepatitis C status and substance use.

Cause of death	HCV-infected PWID			Controls, without HCV^a^, n (%)
	Amphetamine users, n (%)	Opioid users, n (%)	Amphetamine and opioid users, n (%)	
All-cause mortality	1,957 (30.1%)	1,567 (27.3%)	983 (32.0%)	6,671 (4.4%)
Infection	114 (1.8%)	80 (1.4%)	54 (1.8%)	67 (0.0%)
HIV	33 (0.5%)	28 (0.5%)	23 (0.7%)	34 (0.0%)
Neoplasms	260 (4.0%)	112 (2.0%)	71 (2.3%)	1,975 (1.3%)
Diabetes mellitus	32 (0.5%)	15 (0.3%)	13 (0.4%)	129 (0.1%)
Mental and behavioral disorders	128 (2.0%)	127 (2.2%)	91 (3.0%)	145 (0.1%)
Circulatory system	306 (4.7%)	122 (2.1%)	85 (2.8%)	1,452 (1.0%)
Ischemic heart disease	152 (2.3%)	43 (0.7%)	33 (1.1%)	810 (0.5%)
Cerebrovascular disease	52 (0.8%)	22 (0.4%)	16 (0.5%)	244 (0.2%)
Respiratory system	69 (1.1%)	61 (1.1%)	40 (1.3%)	220 (0.1%)
Digestive tract	156 (2.4%)	63 (1.1%)	42 (1.4%)	254 (0.2%)
Glomerular disease	1 (0.0%)	1 (0.0%)	1 (0.0%)	2 (0.0%)
Renal failure	4 (0.1%)	4 (0.1%)	2 (0.1%)	10 (0.0%)
External causes	587 (9.0%)	743 (12.9%)	443 (14.4%)	930 (0.6%)
Suicide	134 (2.1%)	88 (1.5%)	67 (2.2%)	465 (0.3%)
Accidents	333 (5.1%)	469 (8.2%)	276 (9.0%)	420 (0.3%)
Homicide	32 (0.5%)	14 (0.2%)	10 (0.3%)	42 (0.0%)
Groups of special interest				
Liver-related	295 (4.5%)	125 (2.2%)	90 (2.9%)	190 (0.1%)
HCC	98 (1.5%)	31 (0.5%)	28 (0.9%)	31 (0.0%)
Hepatic decompensation	21 (0.3%)	9 (0.2%)	9 (0.3%)	27 (0.0%)
Drug-related^b^	607 (9.3%)	851 (14.8%)	501 (16.3%)	140 (0.1%)
Alcohol-related^b^	454 (7.0%)	207 (3.6%)	179 (5.8%)	655 (0.4%)

^a^ Without amphetamine/opioid use.

^b^ Multiple cause of death.

Abbreviations: HCV, hepatitis C virus; PWID, people who inject drugs; HCC, hepatocellular cancer.

### All-cause mortality

There was no significant difference in crude all-cause mortality between amphetamine users and opioid users, but in the adjusted analysis, the MR was significantly lower among amphetamine users (adjusted mortality rate ratio (aMRR) 0.78, 95% CI 0.73–0.84). The adjusted all-cause mortality was also significantly lower among amphetamine users than among amphetamine-opioid users (Table 3). When compared with controls, the all-cause crude mortality rate ratio (cMRR) and aMRR were high among all groups of PWID, with the highest aMRR among opioid users (aMRR 5.27, 95% CI 4.89–5.68). Crude and adjusted MRRs for all-cause and cause-specific mortality are presented in Table 3.

**Table 3:** Mortality rate ratio.

Cause of death	Amphetamine users^a^ vs opioid users^a^		Amphetamine users^a^ vs amphetamine and opioid users^a^		Amphetamine users^a^ vs controls^b^		Opioid users^a^ vs controls^b^		Amphetamine and opioid users^a^ vs controls^b^	
	cMRR (95% CI)	aMRR^c^ (95% CI)	cMRR (95% CI)	aMRR^c^ (95% CI)	cMRR (95% CI)	aMRR^c^ (95% CI)	cMRR (95% CI)	aMRR^c^ (95% CI)	cMRR (95% CI)	aMRR^c^ (95% CI)
All-cause mortality	0.94 (0.88–1.01)	0.78 (0.73–0.84)	0.90 (0.84–0.97)	0.83 (0.77–0.90)	7.43 (7.08–7.81)	3.44 (3.20–3.72)	7.89 (7.47–8.33)	5.27 (4.89–5.68)	8.23 (7.72–8.78)	4.21 (3.84–4.63)
Infection	1.08 (0.81–1.43)	0.75 (0.56–1.00)	0.96 (0.69–1.32)	1.01 (0.73–1.41)	43.12 (31.89–58.29)	19.45 (12.96–29.20)	40.10 (29.00–55.46)	26.84 (17.88–40.28)	45.00 (31.48–64.34)	18.03 (11.05–29.41)
HIV	0.89 (0.54–1.47)	x ^f^	0.65 (0.38–1.11)	x ^f^	24.59 (15.23–39.71)	x ^f^	27.66 (16.77–45.62)	x ^f^	37.77 (22.27–64.08)	x ^f^
Neoplasms	1.75 (1.41–2.18)	1.13 (0.91–1.42)	1.66 (1.28–2.16)	1.14 (0.88–1.49)	3.34 (2.94–3.79)	2.26 (1.93–2.66)	1.91 (1.58–2.30)	1.93 (1.57–2.36)	2.01 - (1.59–2.54)	1.77 (1.36–2.31)
Diabetes mellitus	1.61 (0.87–2.98)	x ^f^	1.12 (0.59–2.13)	x ^f^	6.29 (4.27–9.26)	x ^f^	3.91 (2.29–6.67)	x ^f^	5.63 (3.18–9.95)	x ^f^
Mental and behavioral disorders	0.76 (0.59–0.97)	0.72 (0.56–0.92)	0.64 (0.49–0.84)	0.69 (0.52–0.91)	22.37 (17.63–28.38)	5.76 (3.66–9.06)	29.42 (23.16–37.36)	10.34 (6.73–15.89)	35.04 (26.94–45.58)	8.55 (5.29–13.81)
Circulatory system	1.89 (1.54–2.33)	1.38 (1.12–1.71)	1.63 (1.29–2.07)	1.22 (0.96–1.55)	5.34 (4.73–6.04)	2.22 (1.83–2.68)	2.82 (2.35–3.39)	1.97 (1.57–2.46)	3.27 (2.63–4.06)	1.74 (1.33–2.27)
Ischemic heart disease	2.67 (1.90–3.73)	1.75 (1.24–2.48)	2.09 (1.44–3.04)	1.41 (0.97–2.05)	4.76 (4.00–5.65)	1.76 (1.36–2.26)	1.78 (1.31–2.42)	1.18 (0.84–1.66)	2.28 (1.61–3.22)	1.14 (0.77–1.69)
Cerebrovascular disease	1.78 (1.08–2.93)	1.33 (0.80–2.18)	1.48 (0.84–23.81)	1.20 (0.68–2.12)	5.40 (4.01–7.28)	3.28 (2.10–5.13)	3.03 (1.96–4.68)	2.76 (1.65–4.61)	3.66 (2.21–6.07)	2.66 (1.40–5.03)
Respiratory system	0.85 (0.61–1.20)	0.62 (0.43–0.88)	0.78 (0.53–1.16)	0.57 (0.38–0.85)	7.95 (6.07–10.41)	3.41 (2.18–5.34)	9.31 (7.02–12.36)	6.49 (4.25–9.90)	10.15 (7.26–14.21)	5.67 (3.45–9.32)
Digestive tract	1.87 (1.40–2.50)	x ^f^	1.68 (1.20–2.36)	x ^f^	15.56 (12.75–18.99)	1.47 (1.16–1.86)	8.33 (6.33–10.97)	1.49 (1.09–2.04)	9.24 (6.67–12.79)	1.01 (0.71–1.45)
Glomerular disease	0.76 (0.05–12.05)	x ^f^	0.45 (0.03–7.25)	x ^f^	12.67 (1.15–139.72)	x ^f^	16.79 (1.52–185.19)	x ^f^	27.92 (2.53–308.00)	x ^f^
Renal failure	0.76 (0.19–3.02)	x ^f^	0.91 (0.17–4.95)	x ^f^	10.14 (3.18–32.32)	x ^f^	13.43 (4.21–42.84)	x ^f^	11.17 (2.45–50.97)	x ^f^
External causes	0.60 (0.53–0.66)	0.60 (0.53–0.67)	0.60 (0.53–0.68)	0.62 (0.55–0.71)	15.99 (14.42–17.74)	9.48 (8.17–11.00)	26.83 (24.36–29.55)	17.85 (15.67–20.34)	26.60 (23.78–29.77)	14.89 (12.67–17.50)
Suicide	1.15 (0.88–1.50)	x ^f^	0.91 (0.68–1.22)	x ^f^	7.30 (6.02–8.85)	2.98 (2.19–4.06)	6.36 (5.06–7.98)	3.25 (2.38–4.43)	8.05 (6.23–10.39)	3.09 (2.12–4.50)
Accidents	0.54 (0.47–0.62)	0.54 (0.47–0.62)	0.55 (0.47–0.64)	0.57 (0.48–0.67)	20.09 (17.40–23.20)	12.33 (10.07–15.09)	37.50 (32.87–42.79)	25.61 (21.51–30.50)	36.70 (31.55–42.68)	21.42 (17.28–26.55)
Homicide	1.72 (0.92–3.24)	x ^f^	1.45 (0.71–2.95)	x ^f^	19.31 (12.19–30.58)	x ^f^	11.20 (6.11–20.50)	x ^f^	13.30 (6.67–26.49)	x ^f^
Groups of special interest										
Liver-related	1.78 (1.45–2.19)	1.03 (0.83–1.27)^e^	1.49 (1.18–1.88)	1.13 (0.89–1.44)^e^	39.35 (32.81–47.19)	5.78 (4.18–7.98)	22.10 (17.65–27.67)	6.23 (4.40–8.84)	26.46 (20.62–33.95)	4.81 (3.32–6.97)
HCC	2.39 (1.60–3.57)	x ^f^	1.59 (1.05–2.41)	x ^f^	80.11 (53.51–119.91)	x ^f^	33.59 (20.43–55.22)	x ^f^	50.44 (30.29–83.98)	x ^f^
Hepatic decompensation	1.76 (0.81–3.85)	x ^f^	1.06 (0.49–2.31)	x ^f^	19.71 (11.15–34.85)	x ^f^	11.20 (5.27–23.80)	x ^f^	18.61 (8.76–39.56)	x ^f^
Drug-related^d^	0.54 (0.49–0.60)	0.53 (0.48–0.59)	0.55 (0.49–0.62)	0.56 (0.50–0.63)	109.87 (91.42–132.04)	89.33 (72.71–109.74)	204.15 (170.69–244.17)	171.81 (141.48–208.64)	199.84 (165.72–240.98)	156.31 (126.05–193.84)
Alcohol-related^d^	1.66 (1.40–1.95)	1.34 (1.13–1.58) ^g^	1.15 (0.97–1.37)	0.93 (0.78–1.11) ^g^	17.57 (15.59–19.79)	16.53(14.65–18.66) ^g^	10.61 (9.08–12.41)	12.32 (10.52–14.42) ^g^	15.26 (12.94–18.00)	17.70 (14.94–20.97) ^g^

^a^HCV-infected.

^b^Without HCV and amphetamine/opioid use.

^c^Adjusted for age, sex, diabetes mellitus, HIV, and alcohol-related diagnosis.

^d^ Multiple causes of death.

^e^ In the adjusted analysis age group 1 and 2 are joined to overcome numerical difficulties caused by no observed deaths in age group 1.

^f^ No reliable adjusted analysis possible due to not enough observed events.

^g^ Not adjusted for alcohol-related diagnosis.

Abbreviations: cMRR, crude mortality rate ratio; aMRR, adjusted mortality rate ratio; CI, confidence interval; HCV, hepatitis C virus; HCC, hepatocellular cancer.

### Liver-related mortality

The crude analysis demonstrated a significantly higher liver-related mortality rate among amphetamine users compared with opioid users (cMRR 1.78, 95% CI 1.45–2.19). However, in the adjusted analysis, there was no longer a significant difference (aMRR 1.03, 95% CI 0.83–1.27), mainly due to adjustment for age and alcohol since the amphetamine users were older and had a higher proportion of alcohol-related diagnosis. Similarly, compared to amphetamine-opioid users, there was a significantly higher rate of liver-related mortality in crude analysis, but not in adjusted analysis. Liver-related mortality was associated with an alcohol-related diagnosis (aMRR 3.16, 95% CI 2.51–3.98), older age (aMRR 40–49 years 8.61, 95% CI 4.93–15.03; aMRR 50–59 years 25.86, 95% CI 15.03–44.51; aMRR 60–69 years 46.66, 95% CI 26.68–81.61; aMRR 70+ years 38.7, 95% CI 16.26–92.05, where age 0–39 years was the reference group), diabetes mellitus (aMRR 1.80, 95% CI 1.44–2.24), HIV co-infection (aMRR 1.66, 95% CI 1.05–2.63), and male sex (aMRR 1.60, 95% CI 1.26–2.02) in HCV-infected PWID.

Compared with controls, liver-related cMRRs were very high across all groups of PWID: 39.35 (95% CI 32.81–47.19) for amphetamine users, 22.10 (95% CI 17.65–27.67) for opioid users, and 26.46 (95% CI 20.62–33.95) for amphetamine-opioid users. With adjustment for age, sex, diabetes mellitus, HIV co-infection, and alcohol, the ratios were lower than in crude analysis: aMRR 5.78 (95% CI 4.18–7.98), 6.23 (95% CI 4.40–8.84), and 4.81 (95% CI 3.32–6.97) respectively.

### Drug-related mortality

Amphetamine users had a lower rate of drug-related death compared with opioid users (cMRR 0.54, 95% CI 0.49–0.60; aMRR 0.53, 95% CI 0.48–0.59) and compared with amphetamine-opioid users. Crude MRR and aMRR were high across all groups of PWID compared with controls (Table 3).

Similarly, death from mental and behavioral disorders (including some ICD codes also used for drug-related mortality) was lower among amphetamine users compared with opioid users and with amphetamine-opioid users, and the mortality was higher across all groups of PWID compared with controls.

In addition, the rates of external causes of death were also lower among amphetamine users compared with opioid users (cMRR 0.60, 95% CI 0.53–0.66; aMRR 0.60, 95% CI 0.53–0.67) and compared with amphetamine-opioid users. The rates were also high across all groups of PWID compared with controls with the highest ratios for opioid users (cMRR 26.83, 95% CI 24.36–29.55; aMRR 17.85, 95% CI 15.67–20.34).

### Alcohol-related mortality

Alcohol-related mortality was higher among amphetamine users than opioid users (cMRR 1.66, 95% CI 1.40–1.95; aMRR 1.34, 95% CI 1.13–1.58), but there was no significant difference between amphetamine users and amphetamine-opioid users. The rates were high across all groups of PWID compared with controls, for amphetamine users cMRR was 17.57 (95% CI 15.59–19.79) and aMRR 16.53 (95% CI 14.65–18.66).

Mortality from digestive tract disorders (including liver disease) was high compared with controls, especially in the amphetamine group. However, it was not as high in the adjusted analysis which was mainly due to adjustment for alcohol. Mortality from neoplasms (including HCC), as well as from infection (including hepatitis and HIV), from diabetes mellitus, and from the respiratory system, was also higher across all groups of PWID compared with controls. Death from cardiovascular disease was higher in all groups of PWID compared with controls and amphetamine users had higher mortality rates than opioid users (cMRR 1.89, 95% CI 1.54–2.33; aMRR 1.38, 95% CI 1.12–1.71).

### Proportions of death and MRR in different age categories

**Fig 2:**
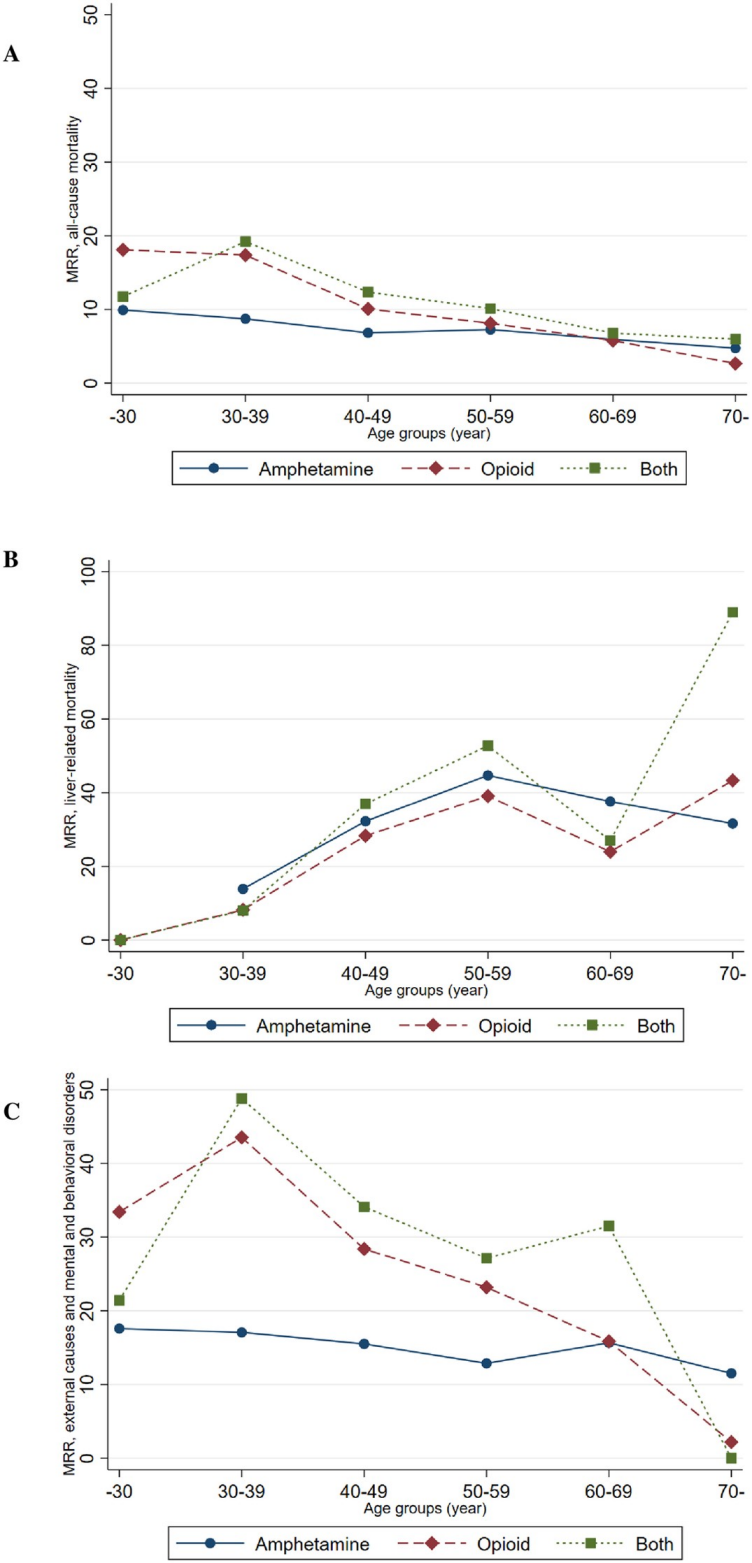
Mortality rate ratio. Hepatitis C virus-infected people who inject drugs compared with matched controls without hepatitis C diagnosis from the general population. (A) All-cause mortality, (B) liver-related mortality, (C) death from external causes and mental and behavioral disorders.

The proportions of death by age groups among amphetamine users are presented in Table 4. Death from external causes and mental and behavioral disorders dominated in younger ages whereas death from neoplasms (including HCC), the circulatory system, and liver-related death was predominant in older age groups. All-cause MRR compared with controls decreased with age (Fig 2A and S2 Table) while liver-related MRR increased with age (Fig 2B and S2 Table). The high MRR in younger age groups was mainly due to death from mental and behavioral disorders and external causes (Fig 2C and Table 4).

**Table 4:** Proportions of deaths by age among amphetamine users.

	-30 years	30–39 years	40–49 years	50–59 years	60–69 years	70- years
	N = 106	N = 261	N = 477	N = 680	N = 368	N = 65
All-cause mortality	106 (100.0%)	261 (100.0%)	477 (100.0%)	680 (100.0%)	368 (100.0%)	65 (100.0%)
Infection	0 (0.0%)	2 (0.8%)	30 (6.3%)	49 (7.2%)	28 (7.6%)	5 (7.7%)
HIV	0 (0.0%)	3 (1.1%)	16 (3.4%)	11 (1.6%)	3 (0.8%)	0 (0.0%)
Neoplasms	1 (0.9%)	7 (2.7%)	35 (7.3%)	112 (16.5%)	85 (23.1%)	20 (30.8%)
Diabetes mellitus	0 (0.0%)	10 (3.8%)	4 (0.8%)	10 (1.5%)	4 (1.1%)	4 (6.2%)
Mental and behavioral disorders	11 (10.4%)	28 (10.7%)	37 (7.8%)	32 (4.7%)	17 (4.6%)	3 (4.6%)
Circulatory system	4 (3.8%)	13 (5.0%)	57 (11.9%)	131 (19.3%)	84 (22.8%)	17 (26.2%)
Respiratory system	2 (1.9%)	7 (2.7%)	16 (3.4%)	17 (2.5%)	23 (6.3%)	4 (6.2%)
Digestive tract	1 (0.9%)	6 (2.3%)	33 (6.9%)	83 (12.2%)	33 (9.0%)	0 (0.0%)
External causes	70 (66.0%)	141 (54.0%)	201 (42.1%)	141 (20.7%)	32 (8.7%)	2 (3.1%)
Suicide	20 (18.9%)	35 (13.4%)	44 (9.2%)	31 (4.6%)	4 (1.1%)	0 (0.0%)
Accidents	35 (33.0%)	72 (27.6%)	107 (22.4%)	91 (13.4%)	27 (7.3%)	1 (1.5%)
Homicide	3 (2.8%)	6 (2.3%)	14 (2.9%)	9 (1.3%)	0 (0.0%)	0 (0.0%)
Groups of special interest						
Liver-related	0 (0.0%)	8 (3.1%)	51 (10.7%)	138 (20.3%)	87 (23.6%)	11 (16.9%)
HCC	0 (0.0%)	0 (0.0%)	10 (2.1%)	40 (5.9%)	42 (11.4%)	6 (9.2%)
Hepatic decompensation	0 (0.0%)	0 (0.0%)	4 (0.8%)	11 (1.6%)	6 (1.6%)	0 (0.0%)

Abbreviation: HCC, hepatocellular cancer.

## Discussion

This national register study of HCV-infected amphetamine users demonstrates a high risk of liver-related mortality, with 1.8- and 39-times higher crude risk compared with opioid users and the general population, respectively. An alcohol-related diagnosis was present in almost 60% of amphetamine users, contributing with HCV infection to an additive risk for liver disease. At younger ages, death from external causes and mental and behavioral disorders dominated, but liver-related death was one of the main causes of death at older ages.

When adjusted for age and other defined risk factors, there was no significant difference in liver-related death between amphetamine and opioid users, even though the crude risk was almost twice as high in amphetamine users. Liver-related deaths occurred mostly in those who were older than 50 years, and in this age group, it accounted for one in five deaths among amphetamine users. The cause of this important finding is multifactorial. Probable explanations are that amphetamine users in this study were born almost 10 years earlier than opioid users, and therefore may have lived longer with HCV infection, and that amphetamine users more often than opioid users reach the age when liver complications may occur since opioid users have a higher risk of drug-related death at younger ages. Furthermore, alcohol diagnosis was significantly more common among amphetamine users than opioid users, which may have contributed to the higher liver-related mortality among amphetamine users. Diabetes mellitus was more common in all groups of PWID compared with the general population with the highest prevalence of 10% in amphetamine users. Thus, an additive effect of alcoholic liver disease, metabolic disease, and viral hepatitis seems to be present in an important number of amphetamine users. Hepatitis C treatment uptake was low among PWID in the period we have studied and only one in six diagnosed with HCV infection had received treatment. However, treatment uptake was equally low in amphetamine and opioid users. Thus, different access to treatment probably does not contribute to the observed difference in liver-related mortality between the two groups of PWID.

All-cause mortality among amphetamine users with HCV infection compared with the general population was high, in accordance with previous studies of amphetamine users including both HCV-infected and uninfected [9,13]. The most frequent underlying causes of death among amphetamine users in this study were external causes, death from the circulatory system, and liver-related death. Adjusted MRR compared with the general population was significantly elevated for every specific cause of death with the highest underlying cause specific aMRR for infections (including hepatitis and HIV), external causes, and liver-related death. A high relative risk of death from external causes was also reported in previous studies on people who use amphetamine [9,13].

A previous register study of a national HCV-infected cohort in Sweden reported a higher all-cause standardized mortality ratio (SMR) in the whole cohort than in a subgroup with transfusion-associated HCV infection [19]. In our study, including only HCV-infected people with substance use-related diagnosis, it is probable that the high all-cause aMRRs compared with controls, to a high degree reflect death caused by substance use, as the relative risks of death from external causes and mental and behavioral disorders were very high. This corresponds well with the results in the previous study.

When comparing all-cause MR in amphetamine users with opioid users there was no significant difference in the crude analysis, but in the adjusted analysis, the mortality was lower in amphetamine users. The finding is mostly explained by the higher age of the amphetamine users. Previous studies similarly have presented lower mortality rates among amphetamine users than opioid users [9,12]. The higher rates of death from external causes and mental behavioral disorders among opioid users compared with amphetamine users might explain this.

The risk of death from the circulatory system was high among amphetamine users compared with controls and compared with opioid users. This has been reported in previous studies as a consequence of amphetamine-induced cardiotoxicity [20]. Another contributing factor might be smoking, which has been reported to be common among amphetamine users [15]. In the present analysis, we did not have access to data on smoking habits.

The all-cause cMRR for the respective group of PWID compared with controls was highest among younger persons and decreased with age. This was mainly explained by the high cMRR of external causes and mental and behavioral disorders in younger ages. Liver-related cMRR increased with age, which may be due to that liver-related consequences from HCV and alcohol mostly emerge after several decades of exposure.

The main causes of death in the amphetamine group were death from external causes and mental and behavioral disorders in younger ages, but in older ages, death from neoplasms (including HCC), the circulatory system, and liver-related death were dominating (Table 4). The finding that drug-related death is dominating in younger ages and liver-related death is one of the main causes in older ages has also been reported in a previous study of HCV-infected PWID, with mainly opioid use disorders [10].

A low proportion had been treated for HCV infection in our study. With new effective and tolerable DAA treatment, liver-related death caused by HCV infection could be prevented. A scale-up of HCV treatment of PWID is also important to reduce HCV transmission and reach the WHO goal of HCV elimination by 2030. The high prevalence of alcohol-related diagnoses among PWID is a challenge that also needs to be addressed.

There are several strengths in this study, especially considering the nationwide coverage of all people registered with an HCV and amphetamine/opioid diagnosis in Sweden, limiting bias from inclusion. The observation time is rather long, which is important when studying chronic HCV infection with complications developing late. Due to the availability of national registers in Sweden, the data have been prospectively collected, eliminating recall bias.

However, our study has some limitations. The date of HCV transmission is unknown since it is common with a delay of several years from transmission to diagnosis. Therefore, the notification date (six months thereafter) was used to set the start of follow-up. As amphetamine users were diagnosed at a higher age than opioid users, delayed diagnosis and lead time bias cannot be excluded.

As HCV notification can be based on either anti-HCV or HCV RNA and both acute and chronic infections are notified to the Public Health Agency of Sweden, some persons included in the study may have cleared their infection, which could have underestimated the risk of liver-related death among those who are infected.

As the SPDR started in July 2005, previous HCV treatment data is missing. However, the treatment uptake in PWID before 2005 has been reported to be very low [21]. Even though we had treatment data from 2005 (Table 1a), we did not have data on sustained viral response (SVR) and were not able to register cured infection after treatment, thereby possibly underestimating the true risk of liver-related death in untreated HCV-infected persons.

Among 52,967 notified cases with HCV infection, 15,317 had a substance use-related diagnosis. In most cross-sectional studies in high-income countries, injecting drug use is the route of transmission in the majority of incident HCV cases. A selection bias can therefore have been introduced. Milder substance use disorders may be present among those without a substance use-related diagnosis. The consequences of HCV infection in this group could thus be different from what we have demonstrated in those with a substance use-related diagnosis.

In addition, individuals with amphetamine diagnosis might have co-used opioids (and vice versa) without it being diagnosed in the registers, resulting in a misclassification. However, existing literature support our results that only a minority of persons with amphetamine/opioid disorders in Sweden co-use the two drugs [15,22].

In conclusion, this national register study presents a higher crude risk of liver-related death among HCV-infected amphetamine users compared with opioid users with HCV infection or the uninfected general population. One in five deaths after the age of fifty was liver-related in amphetamine users with HCV infection. All-cause mortality was higher than in the uninfected population but lower than in opioid users. The higher risk of liver-related death compared with opioid users may be explained by lower competing death risk and higher alcohol consumption. Efforts to increase HCV treatment among amphetamine users, as well as alcohol prevention and treatment, may reduce liver-related death in this group, and implementation and further development of evidence-based treatment for amphetamine use disorder are of great importance for the reduction of all-cause mortality.

## Supporting information

S1 Table Diagnoses/drugs with corresponding ICD/ATC codes. (DOCX)

S2 Table All-cause and liver-related mortality in different age groups. (DOCX)
