# Supplementary material for: Mortality among amphetamine users with hepatitis C virus infection: A nationwide study
Source: PLoS One. 2021 Jun 24;16(6):e0253710. doi: 10.1371/journal.pone.0253710 (PMC8224872; doi:10.1371/journal.pone.0253710)
Supplement: S1 Table — (DOCX) [file pone.0253710.s001.docx]

**S1 Table. Diagnoses/drugs with corresponding ICD/ATC codes**

**Amphetamine use** The National Patient Register (NPR), ICD-9, 304E; ICD-10, F15.x

**Opioid use** NPR, ICD-9, 304A; ICD-10, F11.x

**Alcohol-related diagnosis**

NPR, ICD-9, 291, 291A, 291B, 291C, 291D, 291E, 291F, 291W, 291X, 303, 305A, 357F, 425F, 535D, 571A, 571B, 571C, 571D, 790D, 977D, V79B; ICD-10, E24.4, F10.0, F10.1, F10.2, F10.2A, F10.2B, F10.2X, F10.3, F10.4, F10.5, F10.6, F10.7, F10.7A, F10.7W, F10.8, F10.9, G31.2, G62.1, G72.1, I42.6, K29.2, K70.0, K70.1, K70.2, K70.3, K70.4, K70.9, K86.0, K85.2, O 35.4, Z71.4

**Diabetes mellitus** The Swedish Prescribed Drug Register (SPDR), ATC code A10.x

NPR, ICD-9, 250.x; ICD-10, E10.x-E14.x

**HIV (risk factor)** NPR, ICD-9, 079.J, 279.K, V02.J; ICD-10, B20.x-B24.x, Z21, O98.7; DR, ICD9, 279.5, 279.6; ICD 10, B20.x-B24.x

**Hepatitis C virus treatment**

Direct acting antivirals (DAA)

Simeprevir SPDR, ATC code J05AP05, J05AE14

Daklatasvir SPDR, ATC code J05AP07, J05AX14

Sofosbuvir SPDR, ATC code J05AP08, J05AX15

Dasabuvir SPDR, ATC code J05AP09, J05AX16

Sofosbuvir and ledipasvir SPDR, ATC code J05AP51, J05AX65

Dasabuvir, ombitasvir, paritaprevir and ritonavir

SPDR, ATC code J05AP52, J05AX66

Ombitasvir, paritaprevir and ritonavir

SPDR, ATC code J05AP53, J05AX67

Elbasvir and grazoprevir SPDR, ATC code J05AP54, J05AX68

Sofosbuvir and velpatasvir SPDR, ATC code J05AP55, J05AX69

Sofosbuvir, velpatasvir and voxilaprevir

SPDR, ATC code J05AP56

Glekaprevir and pibrentasvir

SPDR, ATC code J05AP57

Interferon

Peginterferon alfa-2a SPDR, ATC code L03AB11

Peginterferon alfa-2b SPDR, ATC code L03AB10

**Liver transplantation** NPR, ICD-9, 5200, 5201, 5202, 5212, 5214, 5219, 5220, 5221, 5222, 5223, 5224, 5229, 5299, 5280, 5282; ICD-10, JJC00, JJC10, JJC20, JJC30, JJC40, JJC50, JJC60, JJC96, DJ005, DJ006, Z944

**Causes of death**

**Liver-related death**

Viral hepatitis The Cause of Death Register (DR), ICD-9, V02.6, 070x; ICD-10, B15.x-B19.x, B94.2

Liver cancer DR, ICD-9, 155.x; ICD-10, C22.x,

Hepatocellular cancer DR, ICD-9, 155.0, 155.2; ICD-10, C22.0, C22.9x

Alcoholic liver disease DR, ICD-10, K70.x

Hepatic decompensation DR, ICD-9, 456.0, 789.5, 570, 572.2, 572.4, 070.6, 348.3; ICD-10, B19.0, K70.4, K72.x, K76.7, I85.0, I98.3, R18, R18.9, G92.9, G93.4

Other liver diseases DR, ICD-9, 571.x, 572.x, 573.x; ICD-10, K71.x, K73.x, K74.x, K75.x, K76.x, K77.x

**Drug-related death** Based on the variable ”NARKOTIC” in the DR which includes the codes: ICD-9, 304.0-304.9, 965.0, 968.5, 969.6, 969.7; ICD-10, F11, F12, F13, F14, F15, F16, F18, F19, O35.5, P04.4, T40, T43.6, Z50.3, Z71.5, Z72.2.

**Alcohol-related death** Based on the variable ”ALCOHOL” in the DR which includes the codes: ICD-9, 291, 303, 305.0, 357.5, 425.5, 535.3, 571.0-571.3, E860, E980, 980, ICD-10, E24.4, F10, G31.2, G62.1, G72.1, I42.6, K29.2, K70.0–K70.9, K85.2, K86.0, O35.4, P04.3, Q86.0, T51.0–T51.9, Y90.1–Y90.9, Y91.1–Y91.9, Z50.2, Z71.4, Z72.1

**Infection** DR, ICD9, 001–139; ICD 10, A00–B99,

HIV DR, ICD9, 279.5, 279.6; ICD 10, B20-B24

**Neoplasms** DR, ICD-9, 140–239; ICD 10, C00–D48

**Diabetes mellitus** DR, ICD-9, 250; ICD 10, E10-E14

**Mental and behavioral disorders**

DR, ICD-9, 290–319; ICD-10, F00–F99

**Circulatory system** DR, ICD-9, 390–459; ICD-10, I00–I99

Ischemic heart disease DR, ICD-9, 410-414; ICD-10, I20-I25

Cerebrovascular disease DR, ICD-9, 430-438; ICD-10, I60-I69

**Respiratory system** DR, ICD-9, 460–519; ICD-10, J00–J99

**Digestive tract** DR, ICD-9, 520–579; ICD-10, K00–K93

**Glomerular disease** DR, ICD9, 580-583; ICD-10, N00-N08

**Renal failure**  DR, ICD9, 584-586; ICD-10, N17-N19

**External** DR, ICD-9, E800–E999, ICD-10, V01–Y89

**Subgroups of interest**

Suicide DR, ICD-9, E950-E959; ICD-10, X60-X84, Y87.0

Accidents DR, ICD-9, E800-E929; ICD-10, V01–X59, Y85-Y86

Homicide DR, ICD-9, E960-E969; ICD-10, X85-Y09, Y87.1
