# Supplementary material for: Mortality among amphetamine users with hepatitis C virus infection: A nationwide study
Source: PLoS One. 2021 Jun 24;16(6):e0253710. doi: 10.1371/journal.pone.0253710 (PMC8224872; doi:10.1371/journal.pone.0253710)
Supplement: S2 Table — (DOCX) [file pone.0253710.s002.docx]

**S2 Table. All-cause and liver-related mortality in different age groups.**

^a^ Without amphetamine/opioid use

^b^ Since at most one liver-related death occurred among the controls in age group -30, this age group was excluded from the liver-related mortality analysis to circumvent numerical problems in the model parameter estimation.

| Cause of death |  | Age (years) | -30 | 30-39 | 40-49 | 50-59 | 60-69 | 70- | p-value |
| --- | --- | --- | --- | --- | --- | --- | --- | --- | --- |
| All-cause mortality | Amphetamine users vs controls without HCV^a^ | cMRR (95% CI)  aMRR (95% CI) | 9.92  (7.59,12.98)  7.09  (4.76,10.57) | 8.73  (7.40,10.29)  4.54  (3.30,6.23) | 6.82  (6.08,7.64)  2.5  (2.02,3.10) | 7.26  (6.61,7.98)  2.31  (1.97,2.72) | 5.92  (5.28,6.65)  2.53  (2.11,3.04) | 4.75  (3.76,6.00)  2.52  (1.77,3.59) | 0.000  0.000 |
|  | Opioid users vs controls without HCV^a^ | cMRR (95% CI)  aMRR (95% CI) | 18.09  (14.73,22.22)  17.68  (14.09,22.19) | 17.36  (14.81,20.36)  12.58  (10.23,15.48) | 10.07  (8.77,11.56)  5.8  (4.67,7.20) | 8.12  (7.12,9.24)  3.82  (3.14,4.66) | 5.77  (4.82,6.91)  2.53  (1.95,3.28) | 2.65  (1.84,3.82)  1.82  (1.07,3.08) | 0.000  0.000 |
|  | Amphetamine and opioid users vs controls without HCV^a^ | cMRR (95% CI)  aMRR (95% CI) | 11.73  (8.62,15.97)  12.16  (7.95,18.59) | 19.22  (15.53,23.80)  16.89  (12.39,23.02) | 12.35  (10.47,14.58)  5.09  (3.53,7.32) | 10.12  (8.67,11.81)  3.07  (2.24,4.20) | 6.79  (5.30,8.68)  1.67  (1.12,2.50) | 5.96  (3.80,9.33)  2.59  (1.02,6.56) | 0.000  0.000 |
| Liver-related mortality | Amphetamine users vs controls without HCV^a^ | cMRR (95% CI)  aMRR (95% CI) | ^b^ | 13.87  (4.81,39.97)  3.93  (0.28,54.39) | 32.24  (18.63,55.81)  6.63  (2.18,20.21) | 44.69  (31.10,64.21)  6.53  (3.38,12.63) | 37.61  (25.32,55.86)  5.39  (2.74,10.60) | 31.64  (12.78,78.30)  8.51  (1.12,64.75) | 0.000  0.000 |
|  | Opioid users vs controls without HCV^a^ | cMRR (95% CI)  aMRR (95% CI) | ^b^ | 8.22  (1.84,36.72)  1.16  (0.06,22.46) | 28.32  (15.25,52.61)  3.99  (1.57,10.15) | 39.08  (23.32,65.51)  10.34  (3.87,27.63) | 23.96  (11.99,47.87)  4.00  (1.28,12.56) | 43.32  (11.30,166.06)  18.08  (0.61,539.95) | 0.000  0.000 |
|  | Amphetamine and opioid users vs controls without HCV^a^ | cMRR (95% CI)  aMRR (95% CI) | ^b^ | 8.04  (1.80,35.91)  0.63  (0.12,3.40) | 36.94  (16.73,81.57)  3.36  (1.05,10.74) | 52.75  (27.30,101.91)  12.03  (1.82,79.36) | 27.00  (10.66,68.36)  1.70  (0.65,4.44) | 88.93  (10.00,790.52)  28.28  (7.34,108.95) | 0.000  0.000 |
